# Human Life History Evolution Explains Dissociation between the Timing of Tooth Eruption and Peak Rates of Root Growth

**DOI:** 10.1371/journal.pone.0054534

**Published:** 2013-01-14

**Authors:** M. Christopher Dean, Tim J. Cole

**Affiliations:** 1 Cell and Developmental Biology, University College London, Gower Street, London, United Kingdom; 2 MRC Centre of Epidemiology for Child Health, UCL Institute of Child Health, London, United Kingdom; Museo Nazionale Preistorico Etnografico ‘L. Pigorini’, Italy

## Abstract

We explored the relationship between growth in tooth root length and the modern human extended period of childhood. Tooth roots provide support to counter chewing forces and so it is advantageous to grow roots quickly to allow teeth to erupt into function as early as possible. Growth in tooth root length occurs with a characteristic spurt or peak in rate sometime between tooth crown completion and root apex closure. Here we show that in *Pan troglodytes* the peak in root growth rate coincides with the period of time teeth are erupting into function. However, the timing of peak root velocity in modern humans occurs earlier than expected and coincides better with estimates for tooth eruption times in *Homo erectus*. With more time to grow longer roots prior to eruption and smaller teeth that now require less support at the time they come into function, the root growth spurt no longer confers any advantage in modern humans. We suggest that a prolonged life history schedule eventually neutralised this adaptation some time after the appearance of *Homo erectus*. The root spurt persists in modern humans as an intrinsic marker event that shows selection operated, not primarily on tooth tissue growth, but on the process of tooth eruption. This demonstrates the overarching influence of life history evolution on several aspects of dental development. These new insights into tooth root growth now provide an additional line of enquiry that may contribute to future studies of more recent life history and dietary adaptations within the genus *Homo*.

## Introduction

Fossil teeth contain a record of their growth and this can provide insights into the nature of evolutionary change [1]–[3]. Every tooth exists as an individual component within the jaws from which it is partially dissociated both developmentally and functionally. Studying the processes that generate tooth morphology rather than tooth morphology alone is most likely to lead to a better understanding of underlying evolutionary processes [4]. Nonetheless, it also follows that information from many teeth may be required to build a bigger picture of change in dental development through time. One aim of this study was to make measurements of aspects of adult tooth microstructure that can be linked to developmental processes and that can be tracked through time in the hominid fossil record.

Many chronological aspects of primate dental development have now been studied, for example, age at initiation of tooth mineralisation, crown formation times, total tooth formation times and eruption scheduling. Nevertheless, the components of dental development that best reflect changes in primate life history evolution remain unclear [5]–[9] no more so than among hominids (great apes, humans and their extinct relatives). Life history evolution is fundamental to an understanding of how living organisms come to live their lives. An extended period of growth, with both childhood and adolescent phases that may have been essential elements in the evolution of human culture [10], [11], along with reduced posterior tooth and jaw size [12] are among several key attributes that have been used to define the genus *Homo* [13].

Compared with tooth crowns, tooth roots have been relatively understudied but like tooth crowns they preserve evidence of growth processes that reflect both life history evolution and dietary adaptation [14]–[19]. In this study we reconstructed rates of growth in tooth crown and root length in samples of fossil hominid, chimpanzee (*Pan troglodytes*) and modern human teeth. Our primary objective was to investigate the degree of association between peak rates of root growth and the timing of eruption in *Pan troglodytes*, fossil hominids and modern humans and to explore the use of root growth as a means to track the origin and evolution of the extended period of childhood in *Homo sapiens* [10], [11], [20].

Eruption is the process of tooth movement through bone and soft tissue that brings opposing teeth into contact (functional occlusion). First emergence of a tooth through the gingival tissues into the mouth (gingival emergence) is just one late event during this process and occurs before tooth root growth is completed. It is a recordable event that marks the transition between a developmental phase within the bone of the jaw and the beginning of tooth function. The recruitment of each successive opposing pair of teeth into function during growth is advantageous since it increases occlusal stability [21], food processing capacity and energy intake [7], [22]. For this reason attainment of functional occlusion is a biologically significant event and the data for gingival emergence cited here are taken as a proxy for this event.

The mechanisms underlying the eruptive process are not well understood but are likely to be multifactorial [23]–[28]. The initiation of tooth eruption occurs when teeth are still enclosed within their bony crypts. This intraosseous phase of eruption involves the dental follicle and the growth and remodeling of the surrounding alveolar bone. During final reduction of the enamel organ, that overlies the mature enamel cap, proteolytic enzymes are released [25]. These may contribute to the breakdown of the follicle overlying the tooth crown and trigger bone resorption of the roof of the tooth crypt through activation of osteoclasts, perhaps derived from the many monocytes contained within the coronal aspect of the follicle [23], [26], [27], [30]. Mechanisms underlying the supraosseous phase of eruption are, in general, a property of the periodontal ligament (PDL) and its tissues and appear to involve a combination of fibroblast activity and vascular and/or hydrostatic pressure changes within the PDL space [24]. This later stage of the eruption process is, however, also influenced by growth of the jaws [31]. Large numbers of vascular fenestrations exist in PDL capillaries that enable cyclical shifts in proteoglycan and fluid composition [24]. One hypothesis [32] holds that viscous proteoglycan aggregates in the PDL space polymerise and so alter its osmotic pressure, draw in water, and expand so contributing to tooth extrusion and eruption.

Tooth crowns grow in length within the jaws through differentiation of ameloblasts. This begins at the cusp tip and proceeds along the enamel dentine junction (EDJ). Rates of ameloblast differentiation in the tooth cusp are greater than elsewhere and may still be under the influence of the enamel knot, an embryonic signaling centre that defines the position of tooth cusps [33]. The switch to tooth root growth is regulated in the cervical loop of the developing tooth germ through the epithelial stem cell niche of the stellate reticulum [34]–[36]. The end of ameloblast differentiation and of crown formation is associated with repression of FGF-10 and NOTCH signaling and down-regulation of growth factors (such as epidermal growth factor) that sustain the stellate reticulum within the cervical loop [34], [36], [37]. Root growth then continues along the cement dentine junction (CDJ) with differentiation of odontoblasts until completion of the root apex.

## Materials

The samples of teeth used in this study are summarised in Table 1. We sampled only permanent incisors, canines and molars (also abbreviated here and in the Tables and Figures to I1, I2, C, M1, M2, M3). We selected 72 histological sections of modern chimpanzee (*Pan troglodytes*) and 165 of modern human teeth where it was possible to reconstruct growth in tooth length beyond crown formation and as far as possible into the growing root. The modern human sample was primarily selected from a collection made up of individuals of southern African origin that has been described previously [18], [38]. This sample was supplemented with other modern human teeth of diverse geographical and/or archaeological origin. The sample of *Pan troglodytes* teeth was derived partly from a predominantly captive-reared collection of animals described previously [38]–[40] and partly from a predominantly once-free-living museum collection of individuals now housed at University College London. This sample was supplemented with ground sections of additional teeth from once free-living animals housed at the Odontological Collection of the Hunterian Museum, Royal College of Surgeons of England and the Anthropological Institute and Museum, Universität, Zürich. In addition to these teeth we included in our sample 7 impacted maxillary human canines that had been surgically removed from the hard palate (and so had never erupted or come into functional occlusion) and 2 non-erupted, non-functional incisor-like teeth that had been removed from different ovarian tumors (teratomas or dermoid cysts). These were drawn from clinical teaching collections at UCL [41]. We then re-analysed histological sections of four fossil hominid molar teeth previously described in the literature that span the past 18 million years (Table 2). Thus we provide snap-shot examples of dental development from the origin of the first hominids (*Proconsul heseloni* [42], to Late Miocene apes (*Hispanopithecus laietanus* [43] and early hominins (*Australopithecus anamensis*, KNM-KP 30748) [6] and *Homo erectus*, S7–37 [44], [45]. For each of these, some aspects of their enamel and/or dentine growth have already been published. All necessary permits were obtained for the described study, which complied with all relevant regulations.

**Table 1:** Teeth for which growth data appear in Figures 3 and 4.

	*Pan troglodytes*					*Homo sapiens*					Fossils		Other teeth	
	Incis	Can	M1	M2	M3	Incis	Can	M1	M2	M3	M1	M2	Can	Incis
Crown & root	8	13	13	10	10	5	10	15	8	8	2	2	7	2
Root only	9		6		3	39	17	23	20	17				
Crown only						3								
Total	17	13	19	10	13	47	27	38	28	25	2	2	7	2
Total					72					165		4		13
Total														254

**Table 2:** Fossil material represented in Figure 3.

	Locality	Specimen	Geological age	Tooth	Initiation age
*P. heselon*	Rusinga Island	Individual III	17.5–17.9 Myr	M2	0.75
	Kenya ([42]				
*H. laietanus*	Can Llobateres	IPS-1781	9–10 Myr	M1	−0.1
	Spain [43]				
*Au. anamensis*	Kanapoi,	KNM-KP 30748	4.17 Myr	M2	1.75
	Kenya [6]				
*H. erectus*	Sangiran,	S7–37	1.0–1.7 Myr	M1	−0.1
	Java [44], [58]				

## Methods

### Ground Section Preparation

An axial cut was made through the buccolingual longitudinal plane of each tooth crown and root with a diamond wafering blade (*Buehler Isomet Saw*™). One face of the two resulting blocks was polished and fixed with epoxy resin (*Huntsman Araldite 2020*™) to a 1 mm thick glass slide. A second parallel axial cut was then made to remove a thin section still attached to the glass slide. This was then lapped plane-parallel and polished to ∼100 µm thickness, cleared in xylene and mounted with a styrene-based (DPX™) mounting medium for light microscopy. Sections were examined in polarised transmitted light and either digital- and/or photo-montages constructed using x25, x12.5 and x6.3 objectives.

### Incremental Markings in Enamel and Dentine

Circadian clocks exist in ameloblasts and odontoblasts [46], [47] and daily incremental growth markings in enamel and dentine that reflect the periodicity of these clocks make it possible to reconstruct the chronology of tooth development from thin ground sections of fully formed modern or fossil teeth [3], [48]–[55]. Here we used counts and measurements of daily increments in enamel and dentine to put a time scale to growth in tooth length. The thin ground sections of teeth were analysed in transmitted polarised light. Daily growth markings present in enamel and dentine were used to calculate the time taken to form successive increments of tooth length along the enamel-dentine junction and the cement-dentine junction [6], [18], [53], [55].

A record of the orientation of ameloblasts to the EDJ, and of odontoblasts to the CDJ, also persists in longitudinal ground sections of teeth in the form of coarser accentuated incremental markings in enamel and dentine. These can be used to estimate former rates of growth in tooth length because for a given time interval their angulation to the EDJ or CDJ decreases as the rate of differentiation increases during tooth development [55], [56]. These successive measurements of growth rate in tooth crown length can then be cumulated as individual longitudinal growth curves of tooth length against time.

### Estimates of Daily Enamel Rates and Extension Rates

**Figure 1:**
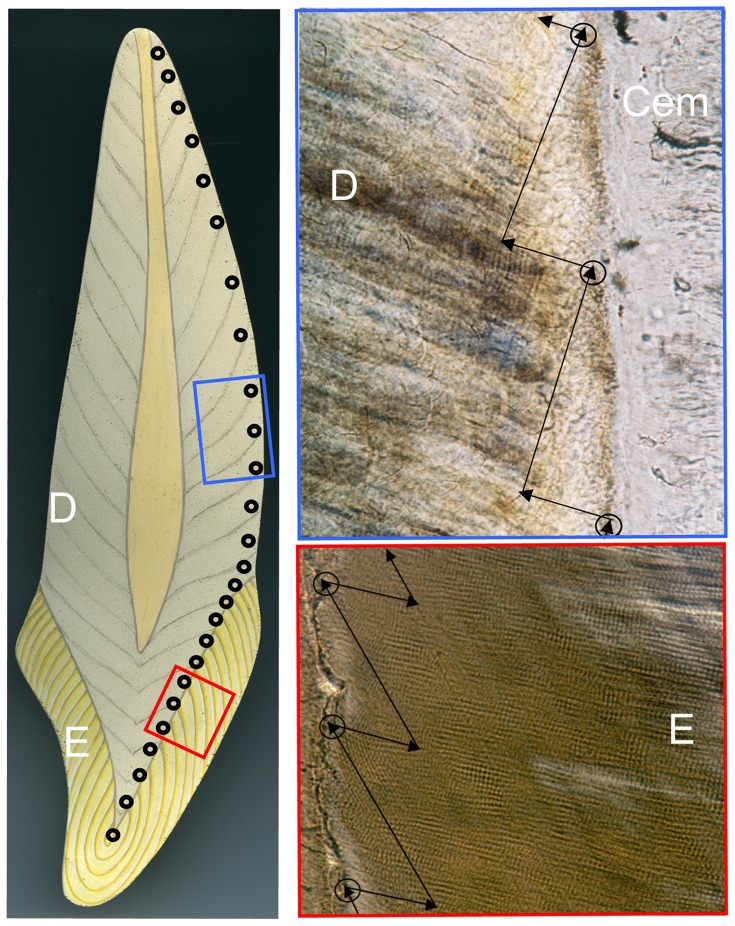
Tooth tissues and their growth increments. Incremental markings within the enamel cap (E) and dentine (D) are depicted on the model tooth (left). The red and blue boxes show details of measurements made along the EDJ and CDJ (from one circle up to the next) using examples of micrographs taken at higher power (right) in which fine daily markings 2–3 µm apart can be seen in enamel and dentine. A thin layer of cementum (CEM) covers the root surface that is not shown in the model.

Figure 1 illustrates a model tooth in longitudinal section with a dentine core (D) (containing a central pulp cavity) and capped with enamel (E). Both dentine and enamel contain regular and widely spaced accentuated incremental markings. Black circles in the small red box represent measurement points along the enamel dentine junction (EDJ). Within the larger red box these circles are superimposed onto a real micrograph of enamel along the EDJ. Prisms of enamel pass from the EDJ, left to right. Daily increments of growth (enamel cross striations) that are ∼2.8 µm apart in this field of view appear as fine cross-hatched markings at right angles to each prism axis. We used previously described methods [43], [57]–[60] where daily enamel increments were counted along the path of enamel prisms from a point at the EDJ (represented by short black arrows running from circles at EDJ) to a second point 200 µm into enamel formation (represented by the head of the short black arrows). From this point, an accentuated marking (also known as a long-period stria of Retzius) was identified running obliquely back to the EDJ within enamel. This stria was then tracked back to intersect the EDJ further along the crown at an end point higher up the EDJ (the direction of these are represented by the long black arrows running obliquely back to the circle above). The distance along the EDJ between the start-point of the short arrow and the end-point at the head of the long arrow represents the length of EDJ formed in the same time it takes to form a 200 µm thickness of enamel. This represents the enamel extension rate that can be expressed in µm/day or µm/year [53], [55]. This procedure was repeated from the cusp tip to the enamel cervix and the data cumulated.

Counts and measurements of daily enamel cross striations were used to determine the time and rate of enamel formation over the 200 µm thickness of enamel defined by this method. These were each repeated in cuspal, lateral and cervical thirds of the same crown. For modern human teeth the average daily rate was between 2.5 to 2.85 µm/day within a 200 µm thickness from the EDJ. Over all these regions the average time to form this thickness was 70 to 80 days. Slower rates (a greater number of daily increments) occurred mostly in the cervical region, especially of M2s and M3s. For *Pan*, counts and measurements of rates were also found to be similar in each region but closer to 3.5 µm/day with an average formation time of 57 days. Slightly lower rates (a greater number of daily increments) were sometimes, but not always, found in cervical enamel of M2s and M3s.

When daily cross striations were not visible, or poorly preserved, an average value was used, calculated using polynomial regression equations [43], [44]. For humans this equation was derived from 20 cuspal enamel prism trajectories (Y = 8.699+0.367×ETµm-0.00005216×ETµm^2^ where Y =  number of days enamel formation and ETµm  =  enamel thickness from the EDJ). The equation gives 80 days as the average time taken to form 200 µm of enamel from the EDJ with upper and lower 95% confidence limits of ∼70 and ∼90 days respectively. For *Pan troglodytes* the equivalent formula based on 12 teeth [30], [31] is Y = 9.391+0.241×ETµm −0.00001556×ETµm^2^. This predicts 57 days as the average time taken to form 200 µm of enamel from the EDJ with upper and lower 95% confidence limits of 51 and 62 days respectively. The higher predicted values compensate for stronger enamel prism decussation (the complex interweaving of enamel prisms as they pass outwards from the EDJ). This often exists, especially in human cuspal enamel but its degree has recently been called into question with new evidence that prisms decussate less than has previously been assumed in regions lateral to the cusp [61]. This supports the use of values closer to 70 or 80 days than 90 days in modern human material but when possible direct counts and measurements of cross striations remain the best way of deciding what number of days to use.

### Estimates of Daily Dentine Rates and Extension Rates

In dentine, regular and widely spaced (typically 15 to 20 µm apart) accentuated markings in root dentine (also described as Owen's lines and/or Andresen lines [62]–[64]) are equivalent to accentuated incremental markings in enamel (striae of Retzius). In longitudinal ground sections of tooth roots they represent a record of the inclination of the former odontoblast cell sheet to the root surface during development. This in turn is a reflection of the rate of proliferation of newly differentiated odontoblasts in the developing root sheath, referred to as the root dentine extension rate that, once again, can be expressed in µm/day or µm/year [55]. In Figure 1 of a model tooth in longitudinal section the black circles in the small blue box represent measurement points along the cement dentine junction (CDJ) at the surface of the tooth root. Within the larger blue box, circles along the CDJ are superimposed onto a real micrograph of dentine covered by a thin layer of cementum (CEM) beneath the root surface (cementum is not represented on the model tooth). The short black arrows indicate the direction of dentine formation. Daily increments of dentine growth (also known as von Ebner's lines) appear as fine-layered markings. We used the method described previously [43], [57], [58] to estimate the time taken to form a 200 µm thickness of root dentine (represented by the length of the short back arrow). Then an accentuated incremental marking was identified at a point 200 µm deep into root dentine (the head of the short arrow) and tracked obliquely back to the CDJ at a point further along the root (represented by the next circle higher up the CDJ). The distance along the CDJ between the start-point of the short arrow and the end-point at the head of the long arrow represents the length of CDJ formed in the same time it takes to form a 200 µm thickness of dentine. This procedure was repeated along the length of the root as far as possible. These data for root growth were then cumulated with those for crown growth.

In modern humans, within a 200 µm thickness of root dentine, the grand mean of measurements made over 3 zones of equal thickness (1.8, 2.56 and 2.76 µm) was 2.4 µm/day. In *Pan* the grand mean over 2 zones within a 200 µm thickness of root dentine (2.26 and 2.42 µm) was 2.3 µm/day. On the basis of previous findings [58] and the likelihood that slower more closely spaced increments close to the CDJ will be less easily visualised and under-represented, these mean daily rates were rounded to 90 days per 200 µm zone. Back-calculations made from tetracycline labels of modern human teeth whose temporal spacing was estimated on the basis of long-period Andresen lines [58] suggest some teeth may have average rates of dentine formation as low a 2 µm/day within the 200 µm zone adjacent to the CDJ. With this in mind, when the last estimate of extension rate in the enamel differed from the first estimate of extension rate in root dentine by more than 1 µm/day the most cervical increments in enamel were re-examined and used to calibrate dentine increments forming at the same time to a value either more or less than 2.3 µm/day. In the fossils previously analysed [65] and represented again in this study the mean daily rates of root dentine measured were 2.4 µm/day for *P. boisei* (KNM-ER 1817, [6], [65], which was used in this study as a proxy rate for *Au. anamensis* (KNM-KP 30748), 2.3 µm/day for *Homo erectus* (S7–37 [65], 2.1 µm/day for *Hispanopithecus laietanus* [43] and 2.0 µm/day for *Proconsul heseloni* [42].

**Figure 2:**
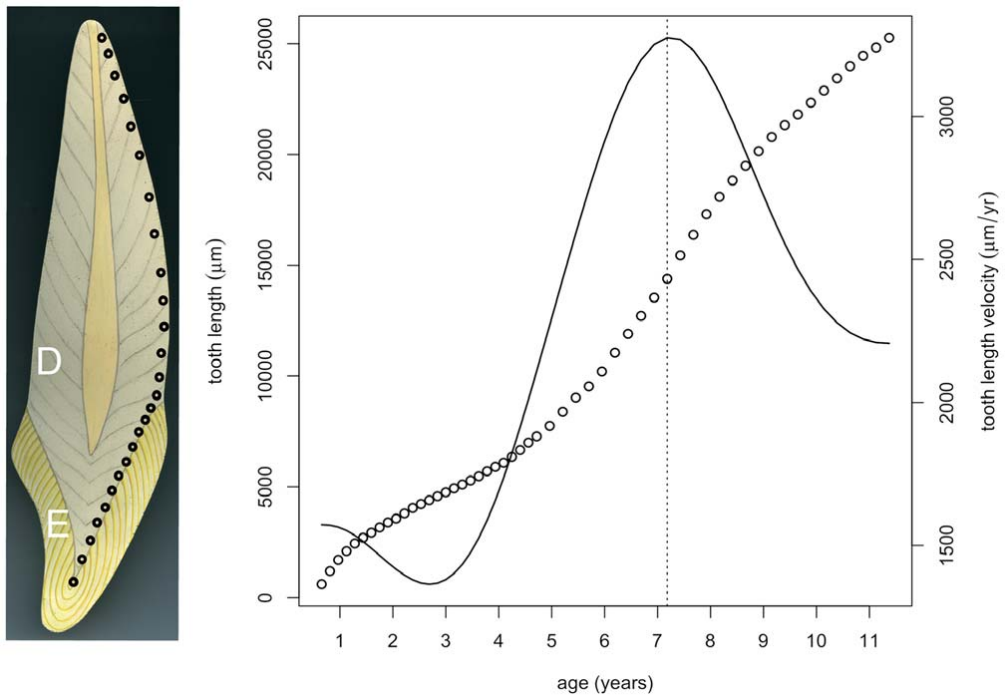
Growth in tooth height. Tooth enamel (E) and dentine (D) both contain widely spaced, obliquely orientated, long-period growth lines. The distance between successive points where growth lines intersect the EDJ or root surface (bold open circles) can be measured in longitudinal thin sections of teeth (Figure 1). Counts of much smaller daily incremental markings between long-period growth lines provide a time scale for successive segments of tooth length (Figure 1). The growth rate is shown by the velocity curve obtained by differentiating a cubic smoothing spline curve through the points. Teeth show a characteristic pattern of growth in length. Growth rates are initially high but then slow towards the end of enamel formation before rising again to a peak in the root dentine. Thereafter, rates reduce again as tooth root formation approaches completion. Approximately twice as many data points appear on the scatterplot as are represented in the simplified model tooth depicted on the left (bold open circles).

These data sets for each individual tooth studied were then cumulated between initial tooth mineralisation in the crown to as far as possible into root dentine growth. Individual distance curves were then plotted each representing a continuous longitudinal record of growth in tooth length along the EDJ through crown and on along the CDJ into root formation (Figure 2).

### Tooth Initiation Times

The cumulated growth data collected for each tooth are a past longitudinal record of their growth and so each can be reconstructed and plotted as distance curves of increasing tooth length along the EDJ/CDJ. Because for isolated teeth chronological age at the start of tooth mineralisation (tooth initiation) can never be known, each tooth of a given tooth type was assumed to initiate at the same point in time. For M1s and anterior teeth this is either 4–6 weeks (−0.1 years) prior to birth or within a few months after birth in both *Pan* and modern humans (0.25 to 0.5 years for permanent incisors and canines). For M2s and M3s these times are both more variable and occur at greater ages so average times are harder to estimate. The tooth initiation times for modern human teeth that have been reviewed and summarised previously [39] were used here; incisors, 0.25 yr; canines, 0.5 yr; M1, −0.1 yr; M2, 3.0 yr and M3, 8.0 yr. For *Pan troglodytes* the mean histological estimates of initiation times in given previously [6], [38]–[40] were used here; incisors, 0.25 yr; canines, 0.5 yr; M1, −0.1 yr; M2, 1.75 yr; M3, 3.69 yr. For fossil molars used in this study (Table 2) the initiation time for M1s was taken to be 0.1 year prior to birth. For M2, initiation times were taken from the literature (*Proconsul heseloni*, [40], [66] and *Australopithecus anamensis* [6].

### Eruption Ages in Fossil Specimens

Gingival eruption can never be defined or observed in fossil specimens and estimates of the timing of this event depend on evidence that it was either immanent or had already occurred [8]. One basic difference between observations about the timing of gingival emergence in living animals and those made on fossils is that the only physical evidence that gingival emergence had certainly occurred in a fossil specimen is cuspal wear, whereas in living primates, for example, a cusp tip just emerged through the gingivae may often be observed long-prior to functional occlusion and well ahead of tooth wear [67]–[70]. Paradoxically, good data for the timing of first functional occlusion in living primates is very difficult to obtain but represents the event of greatest biological significance. Estimates of eruption ages in fossil specimens have been given as ∼3.5 years for M2 in *P. heseloni* [40], [42], 3.4–3.7 years for M1 in *H. laietanus* [43], ‘towards the lower end of the range 5.6–7.8 years’ for M2 in *Au anamensis* [6], at ∼4.4 yr for M1 and at ∼7.6 years for M2 in *Homo erectus* [45].

### Testing the Histological Methods

Only a few teeth in this study could be studied to root apex completion but 90 (51 *Pan*, 39 *Homo*) had clear enough accentuated markings in root dentine to reach ‘Stage G’ as defined by Demirjian et al. (in ref. [71]) or the near-equivalent stage of ‘Root Length Complete’ as defined by Moorrees et al. (in ref. [72]) for modern humans (this being equivalent also to ‘Stage 7’ defined for *Pan troglodytes* by Kuykendall, (in ref. [73]). As a test of the degree of comparability and of potential cumulative error in these histological methods, the mean chronological age for teeth judged to be within ‘Stage G’, ‘Root ¾’ (or ‘Stage 7’) of formation calculated in this study were compared with similar data for the same stage observed radiographically for larger samples of modern humans and captive chimpanzees of known age. These data appear in Table 3. There were no general trends that could be attributed to consistent error in over- or under-estimation of age by the histological technique or to the attribution of ages at initiation used in this study. There was a tendency towards greater chronological age estimates using histology than in the longitudinal or cross-sectional radiographic studies of both known-age modern humans and captive chimpanzees. Given the difficulties of defining stages of tooth formation accurately in any study of dental development and given the small numbers of each tooth type available for comparison in this study these estimates of comparability were considered acceptable. Other tests of histological methods based on individual specimens of great apes [74] of known age and on archaeological human material of known age [75] have reported a difference in their results of between 2 and 5% of the known age at death.

**Table 3:** Ages at stages of root completion in mandibular teeth (years) compared.

	Root stage		I1	I1/I2	I2	Can	M1	M2	M3
*Homo* this study	Stage G	mean		7.75		10.46	7.91	12.1	17.6
		S.D.		1.31		1.35	1.24	1.49	1.63
		n		3		7	13	8	8
*Homo* [76]	Stage 11	mean	7.3		8.1	10.9	7.8	12.5	17.4
		S.D.	0.61		0.61	0.99	0.68	1.11	0.75
		n	121		121	121	121	121	121
*Homo* [77]	R3/4	mean	7.0		8.0	10.2	5.8	11.3	16.3
		S.D.	0.75		0.84	1.06	0.64	1.16	1.67
		n	48		48	136	48	136	136
*Homo* [78,79**†**]	Stage G	mean	6.95		7.78	11.05	6.8	12.23	17.22**†**
		S.E.	0.10		0.11	0.11	0.11	0.13	0.24**†**
		n	4522		4522	4522	4522	4522	45**†**
*Homo* [80]	Stage G	mean	7.85		8.04	11.13	8.0	14.25	17.74
		S.D.	1.18		1.44	1.44	1.60	1.55	1.61
		n	34		33	29	45	140	145
*Pan* this study	Stage G	mean		8.58		9.51	6.28	9.18	11.09
		S.D.		0.70		1.31	1.16	1.51	0.99
		n		7		13	14	7	11
*Pan* [73,81*]	Stage 7	mean	7.93		8.15	9.32	5.75	8.75	11.0*
	Stage G*	S.D.	0.70		0.84	-	1.12	0.97	10.0–12.0*
		n	7		7	1	27	17	5*

Stage 11 [76], Stage G [71], and Stage “root length ¾” (R3/4) [72]. Data for *Pan* are for Stage G [81] or for Stage 7 [73] (that is equivalent to Stage G in [71]). Data for M3* in *Pan* from [81] are for mode and range. In this study samples of I1 and I2 were combined.

### Statistical methods and non-linear growth curve analysis

The mean growth curve representing each tooth type was then determined by synchronising all the individual curves to a single underlying curve [82]. We used SITAR to determine the underlying growth curve for each tooth type of each taxon. SITAR [83] is a shape invariant growth curve model that summarises a number of individual growth curves as their mean curve by transforming each curve as described below. The name SITAR (SuperImposition by Translation And Rotation) highlights the property of the method that after transformation, all the individual growth curves are similar in shape and superimposed on the mean curve.

The SITAR method is uniquely suited to estimate the ages when growth is at its most rapid, i.e. at the age at peak velocity. For each tooth type, e.g. *Pan* canines, the SITAR method assumes that all the growth curves have the same underlying shape, estimated as a cubic regression spline curve. To make the individual curves match this mean curve they are each transformed in three particular ways; shifting them up/down on the measurement scale, shifting them left/right on the age scale, and stretching/shrinking them on the age scale. The first of these transformations corresponds to the *size* of the tooth, the second to the timing or *tempo* of the growth spurt and hence the age at peak velocity, while the third makes the growth curve steeper or shallower and hence measures *velocity*. These three quantities, size, tempo and velocity, are estimated in a mixed model as random effects for each tooth and expressed relative to the mean. The mixed model simultaneously estimates the shape of the mean regression spline curve. The mean age at peak velocity is estimated by differentiating the mean curve and reading off the age when the velocity is at a peak. The standard deviation of the random effect for tempo measures the variability across individual teeth in the age at peak velocity.

## Results

**Figure 3:**
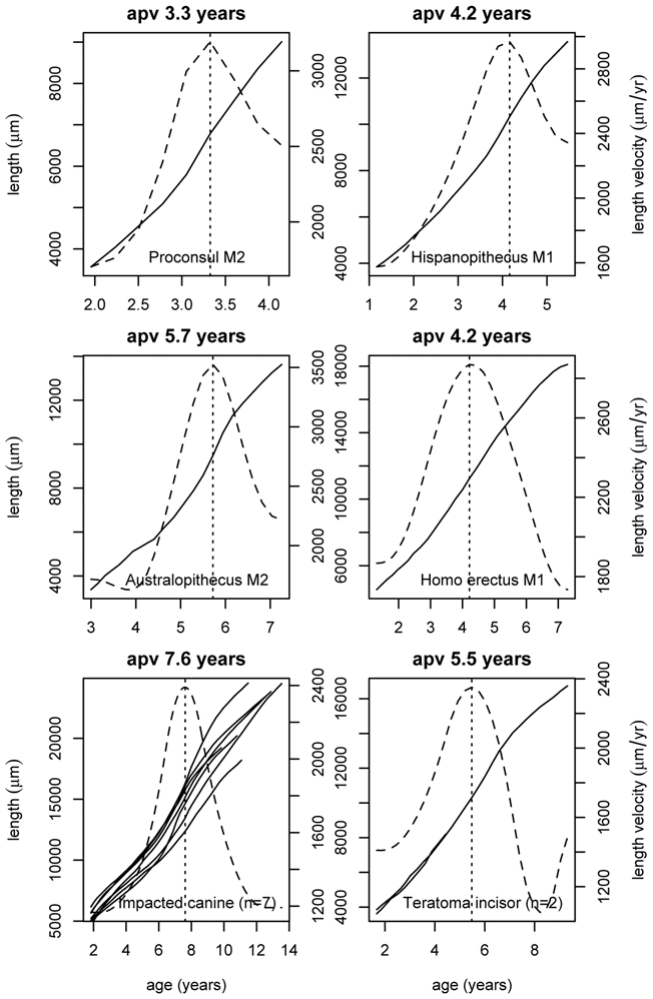
Growth curves for fossil teeth and unerupted human teeth. Reconstructed growth curves, focused here on the root spurt for each tooth, run from bottom left to top right in each plot. Length in μm (left y axis) is plotted against age in years (x axis). Its derivative curve (dashed line) representing growth velocity (in μm/year, right y axis) is superimposed. Age at peak velocity (apv) is shown by the vertical dotted line and the plot title. The fossil molar teeth span the past 18 Mya from one of the first hominoids (*Proconsul heseloni*) to the Late Miocene ape (*Hispanopithecus laietanus*) and early hominins (*Australopithecus anamensis* and *Homo erectus*) and each shows a common pattern of root growth with a clear peak in rate as even do human teeth (bottom row) that never erupted into function.

**Figure 4:**
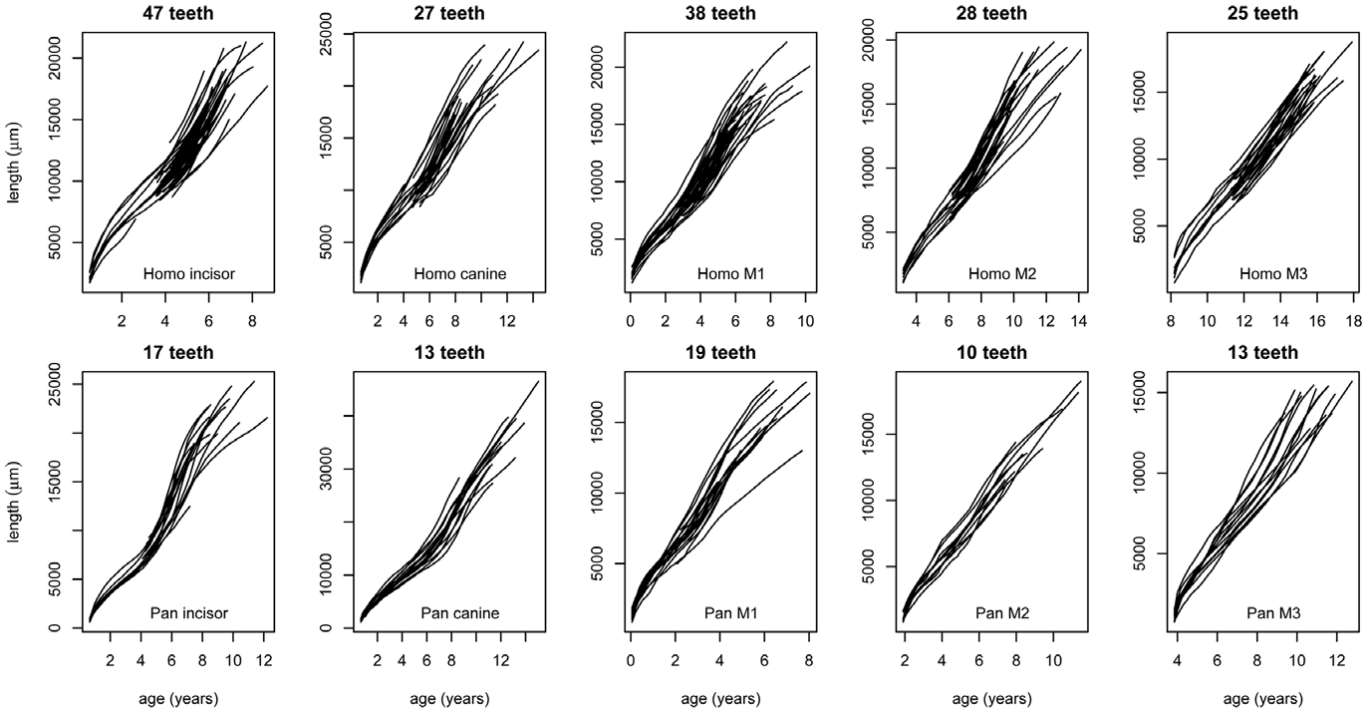
Tooth growth curves for *Pan troglodytes* and modern *Homo sapiens.* Reconstructed growth curves for tooth length. Length in μm (left y axis) is plotted against age in years (x axis) for each tooth type. Average ages for the start of tooth mineralisation were used for each tooth type [6].

**Figure 5:**
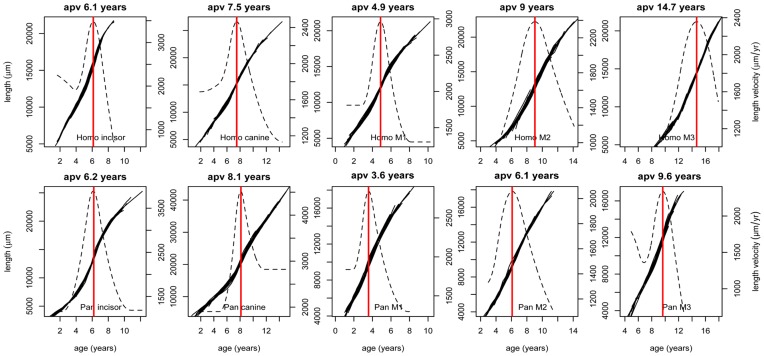
Synchronised growth curves and age at peak velocity. SuperImposition by Translation And Rotation (SITAR) is a shape invariant growth curve model [83] that summarises the individual root growth curves as a single mean curve by transforming each curve so that they are all superimposed. The mean growth curve (bold black) representing the transformed individual curves of each tooth type appears with its derivative (dashed black line) representing root growth velocity (in μm/year, right y axis). Age at peak velocity (apv) is shown by the vertical red line and the plot title.

All teeth studied here showed a common pattern of growth in length (Figure 2). Initially, the growth rate is high in the cusp tips but it then falls, before a second rise and fall in root growth. This appears as a peak in the velocity curve, the age at peak velocity (apv) that is illustrated in Figure 2 where avp occurs at 7.2 years in this model tooth. The isolated fossil molar teeth in Figure 3 also each show a spurt in root growth. The plots in the upper row of Figure 4 show the individual growth curves for each tooth type of *Homo sapiens* and those in the bottom row for *Pan troglodytes*. All teeth of a given tooth type are shown initiating their growth in length at the same time. For both *Pan* and *Homo sapiens* growth in length continues for 8–10 years although few teeth in our sample could be reconstructed to final root apex closure. The plots in Figure 5 show the the original curves after transformation. These correspond to the mean growth curve for the same data as shown in Figure 4. These are represented as continuous bold black lines. Each underlying growth curve is subtly different for each tooth type and must contribute to distinct tooth type morphologies. The dotted lines in Figure 5 represent the mean velocity curves for each tooth type. The vertical red line marks the age at peak velocity. When the red marker lines for apv are compared between *Pan* and *Homo sapiens*, those for molars occur increasingly later in *Homo* but are slightly earlier for canines than in *Pan* but near-equal for incisors. In chimpanzees (*Pan troglodytes*) the average underlying individual ages of the root spurts (Figure 5) correspond closely with the times teeth are erupting into function (M1 ∼3.5 years; incisors ∼6.0 years; M2 ∼6.5 years; canines ∼8 years; M3 ∼10 years [84]–[88] (see also data summarised in Tables 4 and 5).

**Table 4:** Summary of gingival emergence times in captive *Pan troglodytes*.

Age at gingival emergence (in years) for mandibular permanent teeth in captive *Pan troglodytes*									
	Nissen & Riesen [84]			Kraemer et al. [88]		Kuykendall et al. [85]			All captive studies
	from Tables 1A & 2 [84]			from Table 3 [88]		from Tables 5 and 6 [85]			combined
	Total sample n = 15			Total sample n = 17		Total sample n = <58			Total sample n = >90
Tooth	mean	S.D.	range	Med	range	Pr m	S.D.	range	grand mean (range)
I1	5.74	0.62	5.0–7.0	5.60	5.42–5.83	5.46	-	4.81–6.29	5.6 (4.81–7.0)
I2	6.15	0.77	5.0–7.33	5.67	5.42–5.83	5.88	0.26	4.81–6.69	5.91 (4.81–7.33)
Can	8.98	0.76	7.9–10.08	8.17	8.00–8.42	7.96	1.19	6.52–8.74	8.37 (6.52–10.08)
M1	3.27	0.32	2.67–3.75	3.08	2.83–3.33	3.15	0.93	2.14–3.99	3.17 (2.14–3.99)
M2	6.46	0.53	5.58–7.33	6.92	5.83–8.00	6.66	0.63	4.81–6.96	6.68 (4.81–8.0)
M3	10.46	1.45	9.0–13.08	9.92	8.0–11.83	-	-	-	10.19 (8.0–13.08)

Analysis of data for gingival emergence ages in free-living *Pan troglodytes verus* [86], [87] suggests a delay of ∼1 S.D. compared with studies of captive animals reported here [84], [85], [88] with e.g. mandibular M1 emerging at 3^2^/_3_–3^3^/_4_ years [87]. (Med  =  median, Pr m  =  Probit median, S.D.  = 1 standard deviation).

**Table 5:** Gingival emergence times and root apv in *Homo* and *Pan*.

Modern human			Root	Captive *Pan troglodytes*			Root
gingival emergence ages (years)			avp (µm/d)	gingival emergence ages (years)			avp (µm/d)
mandibular	median	±2 SD	Mean (SD)	mandibular	mean	range	Mean (SD)
I1	5.8	4.94–6.66	6.1 (0.4)	I1	5.6	4.81–7.0	6.2 (0.6)
I2	6.8	5.4–8.2	6.1 (0.4)	I2	5.91	4.81–7.33	6.2 (0.6)
Can	9.8	7.78–11.92	7.5 (0.7)	Can	8.37	6.52–10.08	8.1 (0.6)
M1	6.3	5.2–7.4	4.9 (0.6)	M1	3.17	2.14–3.99	3.6 (0.5)
M2	11.4	8.6–14.22	9.0 (1.1)	M2	6.68	4.81–8.0	6.1 (1.1)
M3	18.0	14.0–22.0	14.7 (2.4)	M3	10.19	8.0–13.08	9.6 (1.1)

Summary data for captive *Pan troglodytes* are taken from Table 4 (all captive studies combined). Data for modern humans from [89], [90]. Data for age at peak velocity (apv) from this study. In this study samples of I1 and I2 were combined.

In modern human teeth, as well as those of the fossils and chimpanzees, the same characteristic pattern of growth exists (Figure 4). The growth curves for multiple individual teeth synchronise tightly to an average underlying curve [83] that is characteristic for each tooth type (Figure 5). While in *Pan* the spurts in root growth occur at the ages when the teeth erupt into function, in modern humans, strikingly, they do not. Gingival eruption in modern humans [89] occurs at ∼6 years for incisors, ∼10 years for canines, ∼6 years for M1s, ∼12 years for M2s and ∼18 years for M3s (Tables 4 and 5) but the root growth spurts are earlier than this and occur when the majority of teeth are still contained within the alveolar bone [89].

## Discussion

**Figure 6:**
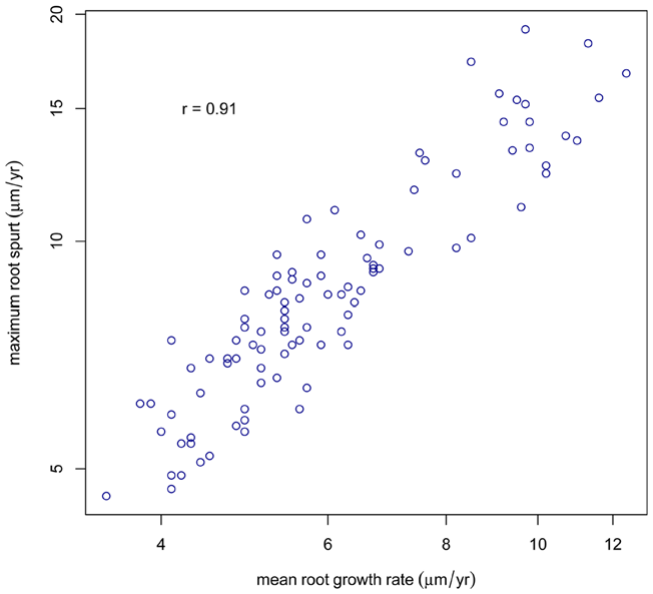
Average and peak rates of root extension. Values, on the y axis, for maximum root extension rates of 100 teeth at Stage G or beyond include those for *Pan, Homo* unerupted teeth and fossils in Figures 3 and 4. These are plotted against the average of all estimates made for rates of root extension in the same tooth (but exclude those estimates made within the root spurt itself).

Previous studies have identified spurts in root growth during tooth growth using measurements made from longitudinal clinical radiographs [72], [89], [91]–[93]. In modern human M1s a marked spurt (with a peak velocity of 18.3 µm/day) was recorded between 4 and 6 years of age [91] and a canine root growth spurt at ∼9 years of age [93] has also been reported (peak velocity 5.5 µm/day). Root growth records in this same study [93] began at 7 years of age at which time rates of premolar root growth were already falling from values greater than 10 µm/day but then interestingly showed a second smaller spurt in growth at 12 to 12.5 years of age prior to apex closure [93]. Histological data for the permanent teeth of *Pan*, *Pongo* and *Gorilla* [18] as well as some fossil hominins and hominids [6], [43] have also previously provided evidence for spurts in tooth root growth. In this study the age at peak velocity for each tooth type is close to those reported previously. It is likely that a number of things may affect the magnitude of the spurt independent of the timing of the spurt. Tooth root shape (discussed below) is one but is difficult to quantify. For 100 teeth in our sample (those for *Pan*, *Homo*, unerupted teeth and fossils shown in Figures 3 and 4) it was possible to calculate an average rate of root extension between the cervix and ‘Stage 7’ [73] or ‘Stage G’ [71] of root formation. This was calculated to excluded the maximum extension rate values within the spurt itself. When average root extension rate is plotted against the maximum extension rate at the spurt for each tooth, it seems the magnitude of the root spurt is to a large degree a function of the average rate of root formation (Figure 6). In general, taller anterior teeth have faster average root extension rates than molar teeth as well as the highest maximum extension rates during their root spurt.

### Root Growth and Tooth Eruption

Root growth rates have been implicated as underlying tooth eruption but also as being the consequence of eruption and not its cause [23]. However, since the process of tooth eruption continues even after surgical resection of the growing root [24], [94], [95] it is clear that eruption is not driven by tooth root growth alone. Nevertheless, since tooth eruption gathers speed towards occlusion [32], [96] there is still an expectation that normal tooth root growth might mirror this. Even if individual variables can be shown experimentally not to be driving the eruption process on their own, there remains a strong possibility that the mechanisms of eruption are multifactorial [23], [24], [26], [27], [32], [94], [96]. Faster eruption rates than root growth rates [96] create space beneath the growing root apex into which the developing apical papillary tissues might proliferate more readily than when root growth rate is equal to or even exceeds the eruption rate [23]. Moreover, it has been demonstrated in modern human subjects that both the amount by which a tooth erupts, as well as the amount of tooth growth in length, is larger in individuals with mandibular bodies of greater vertical height [97], [98]. It follows root growth and tooth eruption may well be influenced by growth of the jaws, and especially by space availability in the jaws [99], [100] even if the initiation of tooth development is not [101]. Furthermore, there is evidence that the factors that control initiation of the intraosseous eruption phase, through bone resorption of the crypt roof, may also influence root diameter but not apparently root length [102].

To demonstrate that there is an independent and underlying genetic component to the control of odontoblast proliferation during tooth root growth, and that the timing of the spurt in root growth is intrinsic to tooth growth and is not just a local environmental phenomenon, we reconstructed rates of root growth in a sample of human unerupted impacted maxillary canines and teeth from ovarian teratomas. These teeth still also showed a spurt in root growth (Figure 3) and at times during tooth formation within the range of our normal human sample.

It has been suggested that gingival emergence ages for free-living chimpanzees may be later than those observed in captivity, although by how much and exactly why this should be remains a topic of debate [8], [84]–[87]. While the timing of the root spurt seems to be an intrinsic component of tooth development (and no evidence exists to suggest it corresponds with eruption into functional occlusion in an individual) it is notable that the underlying average age of the root spurt in our sample of chimpanzee teeth match the mean or modal ages of gingival eruption so closely in completely different samples of living animals reported in the literature. For M1 in *Pan troglodytes*, where gingival eruption ages have perhaps been best reported, age at peak velocity occurs at 3.6 years. This falls slightly closer to the ages reported for gingival emergence in free-living *Pan troglodytes verus* than in captive animals (Tables 4 and 5) but for other tooth types, age at peak velocity fits the captive data better (where samples of individuals observed are larger). It follows that our data for rates of tooth root growth in *Pan troglodytes* suggest that in future even using root growth data collected from small samples of isolated teeth (for example ∼10) in other modern and fossil taxa may prove valuable for reconstructing a likely range of ages of tooth eruption. In fossil taxa especially any means of assessing the pace of general growth makes an important contribution to our understanding of life history evolution.

A key finding in this study is that in modern humans average age at peak velocity in root growth does not correspond with age at gingival emergence or age at eruption into function. One exception among our sample of modern human teeth is incisors. Modern human incisors still show a close correspondence between the timing of their root growth spurt (6.1 years) and their eruption into function at ∼6 years of age. Surprisingly, in all living great apes the average age of incisor eruption is also between 5 and 6 years of age. Indeed, it might well have been close to this in *Australopithecus* [6] and early *Homo* as well [44], [45], [103]. It is also notable that in *Homo erectus*, incisor size has not reduced with respect to that in *Australopithecus* in the way posterior tooth size has [104]. Whatever the dietary and functional implications of this for *Homo erectus*, it may be that space in the growing jaws was just too limited to form large anterior teeth in a shorter time [100]. Consequently, during human evolution as the general growth period has become prolonged, with respect to living great apes, incisors have erupted into function progressively earlier in childhood. The reasons for relatively earlier permanent incisor eruption in modern humans remain obscure but may in part be to do with the maximum functional life span of small deciduous incisors whose size is constrained during development by the size of the fetal jaws. By 6 years of age most deciduous incisors have usually been subjected to considerable wear and tear and would not last for another 6 years in modern humans until M2 eruption under increasing masticatory loads (as they do in great apes). Perhaps for this reason selection has held permanent incisor eruption at a more or less chronological constant during human evolution.

There are a number of possible explanations for the general dissociation between age at peak root velocity and tooth eruption into function among modern human permanent canines and molars. The first supposes that root growth spurts are non-adaptive and bear no relation to the eruption process but rather that spurts in root growth simply reflect or determine some aspect of tooth morphology. Enamel crown formation times, the position and timing of root bifurcation formation or the bulging root surface contours of some teeth might all in theory underlie the timing of the root growth spurt. Given the modular nature of tooth growth within the jaws it is indeed likely that many dental developmental events are tightly integrated and are the basis of distinct tooth type morphologies. Indeed, if the timing of the root spurt were not constrained developmentally, or indeed by directional selection, the expectation in modern humans would be for it to have become far more variable than it is. However, the hypothesis that the timing of the root spurt is linked to tooth morphology in some way weakens on grounds that all teeth seem to show a spurt in root growth whether or not they are single or multi-rooted, or are conical or express some degree of mid-root bulbosity in buccolingual cross-section. When roots first increase in diameter and then reduce again towards the apex, the spurt does not rise and fall over the whole curvature of the root. Rather, it is confined to a smaller section, typically between 2 and 4 mm, of the total root length. Similarly, EDJ contours in the crowns of teeth may be sinuous as they approach the root cervix but still tend to have uniform extension rates that transition across the cervix into root with either a smooth gradient of change in extension rates or often with little or no change at all. No clear association with any one of these variables or with the level of the root furcation in multi-rooted teeth appears to exist between the timing of the root spurt in the teeth sampled here. The best association observed in *Pan troglodytes*, but not in modern humans, remains between the timing of the root spurt and tooth eruption into functional occlusion.

Growth spurts of any kind are more likely to be (or have been) adaptive in some way and more likely mark a transition between one phase of growth and another. In this case, in all but modern human teeth, the root spurt marks a transition between tooth development within the bone of the jaws and functional occlusion within the oral environment. Tooth roots provide support to counter chewing forces and it is clearly advantageous to grow roots quickly to enable teeth to erupt into function and so be able to resist and stabilise masticatory forces as early as possible. After this time there may be less of an imperative to continue to grow roots at the same fast rates.

### Dental Development in Early *Homo*

Surprisingly, the average ages at peak root velocity in modern human teeth come close to the ages estimated for tooth eruption in the few specimens of *Homo erectus* where it has been possible to say something about this. Molar emergence times in *Homo erectus* have been estimated at 4.5 to 5 years for M1 [20], [44], [105], 7.6 to 9 years for M2 [45], [105] and 14.5 years for M3 [105]. These estimates for molar eruption ages in *Homo erectus* have been considered to be intermediate between the mean or modal molar eruption ages typical of living great apes and those of modern humans today [45], [105]. An age at death of between 7.6 and 8.9 years for the Turkana boy (KNM-WT 15000) provides some evidence for the timing of permanent tooth eruption, all be it for a single individual [45]. Newly erupted mandibular canines (with maxillary canines still unerupted) and recently erupted M2s (and premolars) had just come into functional occlusion in this specimen. A near-completed M3 crown in this specimen would put initiation of this tooth ∼2.5 years earlier in *Homo erectus* [44]. KNM-ER 820 is a younger *Homo erectus* specimen where only the crypt for M3 is visible on radiographs and where lower I2s were just coming into function. The developing canines, premolars and M2s were at a stage of development equivalent to those of KNM-WT 15000 at ∼6 years of age if crown formation times and rates of root growth were the same [45]. Evidence for rates of crown formation, crown completion times and root formation rates for M1 and P4 in *Homo erectus* also come from two histological sections of a specimen (S7–37) from Java [44]. These data provide an additional provisional chronological time scale against which each of these specimens can be attributed an age at death [44], [45], [58]. A further specimen attributed to early *Homo*, Stw 151 from Sterkfontein, South Africa, although probably not *Homo erectus*, had also recently erupted permanent incisor teeth at the time of death and has been aged histologically to 5.3 years of age [103].

If, as in *Pan troglodytes*, the average age of the tooth root spurts in *Homo erectus* corresponded closely with the average age of tooth eruption into functional occlusion then the average ages at peak root velocity would be expected to occur at approximately 5–6 years for incisors; 7.6–8.9 years for canines; 4.5–5 years for M1; 7.6–8.9 years for M2; and ∼14.5 years for M3. Only one root spurt in *Homo erectus* is known for the M1 of S7–37 from Java where age at peak root velocity occurred at 4.2 years (Figure 3) but those reported here for modern humans (Figure 5) come closer to these predictions for *Homo erectus* than they do for the known eruption ages in modern humans (with the exception of incisors discussed above). They are as follows: Incisors, 6.1 years; canines, 7.5 years; M1, 4.9 years; M2, 9 years; M3, 14.7 years. (The estimates for molars, it should be noted, assume the same initiation ages for *Homo erectus* as in modern humans, which may in fact have been earlier). These data suggest that the range of ages of peak velocity in tooth root growth that may have existed in *Homo erectus*, or one or other immediate descendant species of *Homo*, may still persist unaltered among modern human teeth today.

### Life History Evolution and Hominin Tooth Root Growth: A Hypothesis

Earlier than expected peak rates of root growth in modern humans can be explained when an extended life history schedule and reduced chewing forces acting on the jaws and teeth are considered together. Smaller tooth crowns in modern humans with longer roots formed than in *Pan, Gorilla* or *Pongo* [106], [107] by the time they enter functional occlusion have relatively greater root surface areas for tooth support despite reduced tooth size and reduced chewing forces [12]. Longer roots formed at eruption are simply the consequence of modern human tooth roots having more time to grow prior to their eruption into function. Selection acting to ensure that sufficient root attachment area develops in time to support teeth as they erupt into function is, therefore, now neutralized. There is no advantage in modern humans for the peak in root growth rate to shift to a later age and so the timing of it persists as an integral component of tooth root growth. Indeed, the signals underlying the spurt may still be linked to, or constrained by, other intrinsic tooth growth processes that are developmentally independent of the eruption process.

If this explanation for the dissociation between the timing of spurts in tooth root growth and the ages at tooth eruption in modern humans is correct, then the timing of the root growth spurt in modern human teeth reflects the last stage in human evolution when it conferred an advantage. In *Homo erectus* the growth period was still short enough [20], [44], [45] and chewing forces at the time of tooth eruption presumably still great enough, to require rapid root growth right up until the time teeth erupted into function. If this were the case, the same timing of the root growth spurt persists now only as a marker event that remains intrinsic to the root growth processes.

All tooth types studied here, both fossil and modern, show a spurt in root growth. In *Pan troglodytes* the timing of the root spurt coincides with tooth eruption into function. In modern humans, with the exception of incisors, the root growth spurt now lags behind and has become dissociated from the later ages of tooth eruption into functional occlusion. Regardless of the adaptive or non-adaptive nature of the root spurt, this demonstrates that root growth spurts in modern humans play no role in the eruptive process. Moreover, the data presented here for root growth show that later canine and molar eruption ages in modern humans have not resulted from slower daily dentine rates or slower root extension rates since these now appear to be little different in *Pan* and *Homo*. Rather, selection has acted independently of tooth tissue formation to delay molar initiation times, but most of all to delay initiation of the eruption process itself.

**Figure 7:**
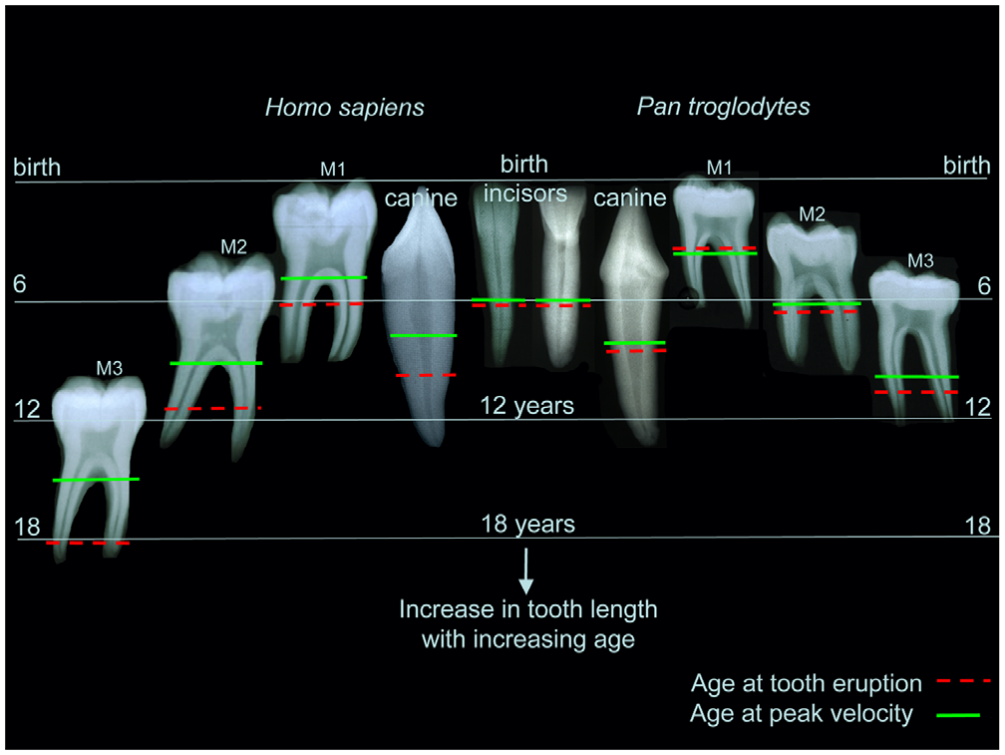
The timing of tooth development in *Pan* and *Homo sapiens*. The sequence of initiation of tooth mineralisation through to tooth root completion is compared for incisors, canines and molars in *Pan* and *Homo*. Time is on the y axis and teeth begin to develop between birth (top line) and ∼12 yr in *Pan* or ∼18 yr in *Homo* (bottom line). Teeth are vertical, as they appear in the lower jaw, and increase in height through time. Tooth size and shape are not to scale. M1 in *Pan* and *Homo* begin to mineralise slightly before birth, but M2 and M3 in *Homo* initiate at later ages than in *Pan*. Consequently, M3 root completion occurs ∼6 yr later in *Homo* than in *Pan*. Molar eruption ages in *Homo* (dashed red lines) are drawn out disproportionately to later ages such that M1, M2 and M3 erupt into functional occlusion with increasingly longer roots formed. As a result, M3 eruption in *Homo* occurs ∼9 yr later than in *Pan*. In *Pan* for all teeth the timing of average age at peak root velocity (green lines) corresponds closely with age of eruption into function, but in *Homo* this has not kept pace with the later eruption ages for canines or molars from which it has become dissociated. Ages for tooth initiation and root completion are mean values from the literature [6], [79], [85].

Not all stages of ages of attainment for tooth formation in *Pan* and modern humans are easily compared in longitudinal radiographic and histological studies of dental development but some are more easily definable than others. Total tooth formation times, that include both enamel crown and root dentine formation times, are surprisingly similar for anterior teeth and overlap in their ranges for molars [6], [73], [79]. By way of example, equivalent ages of attainment of root formation stages in permanent incisors in *Pan* (‘Stage 7’) [73] and modern humans (‘Stage G’) [79] respectively, occur at 7.93 years (S.D., 0.68) and 7.85 years (S.D., 1.0). Our findings, therefore, support the conclusion that age at eruption of molar teeth is a better reflection of the pace of general development in hominids than rates of tooth tissue formation, or total tooth tissue formation times in any one tooth type. These events for *Pan troglodytes* and modern humans are illustrated schematically in Figure 7 where some of the key temporal components of dental development are compared.

Studies of other living and fossil primates are likely to shed more light on the nature and timing of tooth root spurts. Many living primates may well have different life history schedules than many Miocene apes and monkeys [17], [43], [66] and so future comparative work on both extant and fossil primates is a good way of gaining a bigger perspective on tooth growth within the total growth period. Time available to grow teeth, both up until the age of eruption into function but also within the available period of general growth, places a constraint on how long crowns and roots of different sizes may take to form. Deciduous teeth form quickly with very high extension rates of up to 19 µm/day in modern humans but no spurts in deciduous root growth have been reported [108]. Tooth roots in *Gorilla* and *Pongo* are longer (considerably so in molars) than in *Pan* [17] and require higher extension rates to complete root growth in the same or similar time as *Pan* [6], [18]. Root spurts may not, therefore, always exist in teeth where extremely fast extension rates are required to persist throughout the whole period of root formation in order to achieve long root lengths in a short time. On the other hand, the greatest rates of root extension at PHV appear to be associated with fast average rates of root formation (Figure 6) such that both may be an indirect measure of time constraint on tooth formation.

The amount of root formed on molar teeth at eruption increases in both *Pan* and *Homo* from M1 through M2 to M3 (Figure 7) as larger teeth and greater chewing forces at initial occlusion require more PDL surface area to counter this. It has been observed that M3s in *Gorilla* erupt with less root length formed than in *Pan* [109] which may be part of a trend where less M1 root length is formed in *Pan* than *Gorilla* and *Pongo*, a similar M2 root length is formed in all three, but a greater M3 root length is formed at eruption in *Pan* than either *Gorilla* or *Pongo* [106], [109]. In this respect, the drift of mean molar eruption ages in modern *Pan troglodytes* to later ages than the mean ages of the root spurts depicted in Figure 7, may be evidence of an increasing dissociation between the two through the growth period. If confirmed, this may reflect a real difference in dental and/or life history scheduling in living *Pan troglodytes* than in, say *Gorilla* [8]. An alternative explanation is that raising the bifurcation of molars in *Gorilla* closer to the cervix (a morphology many early fossil hominin teeth share) simply increases root surface area by another mechanism than increasing root length at eruption [19]. The ultimate goal of future comparative studies is to contribute to our understanding of how general growth, jaw growth and tooth growth interact with each other and how they do or do not influence the processes that initiate and control the timing of tooth eruption [110], [111].

Tooth root morphology and tooth root growth processes provide clues about general growth and about dietary adaptation in primates. One goal in human evolution is to understand more about the interplay between life history evolution and dietary diversity among the various species of *Homo* during the last 1 million years. The earliest evidence of a modern extended period of growth has been reported in *Homo antecessor* [112] between 0.8 to 0.96 Mya. This predates the time (0.3 to 0.4 Mya) when the controlled use of fire for cooking became widespread and well established in Europe [113]. Similarly, the first occurrence of *Homo erectus* at 1.9 Mya [114] still predates the first clear evidence of fire in Africa at 1 Mya [115]. Yet it is the widespread use of fire for cooking that is held to be most influential in reducing chewing forces, the duration of intra-oral food processing and in reducing posterior tooth and jaw size [12]. Comparative studies of tooth root morphology that track growth changes in length and root surface area through time, alongside changes in enamel thickness, in *Homo erectus, Homo antecessor, Homo heidelbergensis, Homo neanderthalensis* [19,57,116.117] and the earliest fossil remains of modern *Homo sapiens*, may now provide another way of exploring important aspects of dietary and life history evolution during these later stages of hominin evolution.
